# Global Financial Crisis, Smart Lockdown Strategies, and the COVID-19 Spillover Impacts: A Global Perspective Implications From Southeast Asia

**DOI:** 10.3389/fpsyt.2021.643783

**Published:** 2021-09-03

**Authors:** Chunlei Wang, Dake Wang, Jaffar Abbas, Kaifeng Duan, Riaqa Mubeen

**Affiliations:** ^1^School of International Economics and Trade, Shanghai Lixin University of Accounting and Finance, Shanghai, China; ^2^School of Media and Communication, Shanghai Jiao Tong University, Shanghai, China; ^3^Antai College of Economics and Management, Shanghai Jiao Tong University, Shanghai, China; ^4^School of Economics and Management, Tongji University, Shanghai, China; ^5^School of Management, Harbin Institute of Technology, Harbin, China

**Keywords:** COVID-19, public health, economic crisis, smart lockdown strategy, social factors, spillover impacts

## Abstract

This present study primarily emphasizes to seek the COVID-19 adverse impacts posing health challenges and global economic crisis. The pandemic (COVID-19) continues to hit the global economies adversely. Pakistan is the 5th-most-populous nation, and recorded positive cases with the third-highest positivity ratio in South Asia, and 26th-highest deaths toll of 21,450 and 29th number of most COVID-19 positive cases with 933,750 worldwide, as of June 6, 2021. The first wave appeared at the end of May 2020, and mid of June reported its peak, which ended by mid-July 2020. Early November 2020 witnessed the second wave with low intensity reached the climax by mid-December. The COVID-19's third wave severely affected the country during mid-March 2021. It exhibited the highest positivity rate, around 20%. New positive patients and deaths toll commenced to skyrocket and reported peak by April 15, 2021. Then situation gradually improved with effective measures and restrictions. The pandemic coronavirus (COVID-19) has affected 220 territories, regions, and countries and resulted in more than 174.116 million infections, deaths, 3.75 million, and 157.157 million positive cases fully recovered from this infectious disease, as of June 7, 2021. The pandemic has caused a severe crisis of healthcare facilities and economic challenges worldwide. Pakistani economy reported GPD's negative growth (–0.05) for the first time over the last 60 years in 2020, which caused a massive financial crisis. The Government's relief package intervened to reduce public mental stress and improve the quality of their lives. IMF reported that Pakistan's GPD bounced back at 4% growth by June 2021. This article determines that economic instability and health burden happened in Pakistan for a longer time than financial disequilibrium that occurred globally. Pakistan encountered this crisis due to its feeble healthcare systems and fragile economy. This study explores adverse health issues and spillover consequences on the economic crisis in Pakistan with global implications. It recommends smart lockdown restrictions in most affected areas to reopen the economic cycle with strict preventive measures to minimize the COVD-19 adverse consequences.

## Introduction

The advent of coronavirus brought the global health emergency caused by the spread of the novel COVID-19 disease, which affected almost all the countries, including the most developed and advanced nations and the weak economies worldwide (1–5). This global economic crisis has had adverse effects on individuals' quality of life and mental health (6–10). The ongoing coronavirus (COVID-19) pandemic appeared in Wuhan (Hubei region, China), and the first case of infection was reported on December 31, 2019, in the region. The outbreak of the COVID-19 epidemic has caused a global health emergency. The pandemic coronavirus (COVID-19) has affected 220 territories, regions, and countries and resulted in more than 174.116 million infections, 3.75 million deaths, and 157.157 million positive cases fully recovered from this infectious disease, as of June 7, 2021. The emergence of a pandemic (COVID-19) has also hugely affected Pakistan's economy (11–14). Pakistan is the fifth most populous nation and recorded positive cases with the third highest positivity ratio in South Asia, and had the 26th highest deaths toll of 21,450 and 29th most COVID-19 positive cases with 933,750 worldwide, as of June 6, 2021. The first wave appeared at the end of May 2020, reported its peak in mid-June, and ended by mid-July 2020. Early November 2020 witnessed the second wave with low intensity, which reached its climax by mid-December. COVID-19's third wave severely affected the country during mid-March 2021. It exhibited its highest positivity rate, around 20%. New positive patients and death tolls commenced to skyrocket and reported a peak by April 15, 2022. Then the situation gradually improved with effective measures and restrictions. It caused enormous health, economic, environmental, and social problems. In Pakistan, health officials reported 564,824 confirmed infected cases, 12,380 indicating a 2.2% case fatality rate and total recoveries of 527,061, as of February 15, 2021 (15). The findings of a previous study reported that 33–42% of the admitted patients facing Middle Eastern respiratory syndrome (MERS) and SARS-CoV, known as severe acute respiratory syndrome, exhibited various health issues. The patients admitted to hospitals showed depressed mood, anxieties, stress, insomnia, mental distress, and impaired memory. Some recovered patients reported adverse effects of the disease after their recoveries. Consequently, virus infection caused various family issues and increased domestic violence and physical and mental health problems worldwide (16).

This virus' (COVID-19) symptoms vary; nevertheless they most commonly include fever (17), headache, cough (18) fatigue, breathing difficulties, a loss of taste, and a loss of sense of smell (19–22). The virus attack can appear from day one to fifteen or even longer after exposure to the infected person or environment. Research indicated that almost 35% of infected people do not show notable symptoms (22–25). People with noticeable disease symptoms are patients of coronavirus (26, 27). Over 4/5 people (81%) develop noticeable mild-to-moderate health issues, such as pneumonia, and 14% of COVID-19 positive people report severe symptoms, including hypoxia and dyspnea. In addition, 5% of people develop acute symptoms of coronavirus, which results in shock, respiratory failure, or other health issues like multiorgan dysfunction (28). Studies reported that older people face a higher risk of virus attack and developing acute symptoms. Some patients have faced a series of health issues several months after a successful recovery from this (Covid-19) disease (29).

For the first time in 60 years, GDP has shown a negative growth, exacerbating the enormous financial crisis and recession. It has affected the quality of life of the public massively. Self-segregation, social alienation, and travel restrictions have forced the labor force to decline in all sectors of the economy, resulting in unemployment. The whole industry is facing a blockade, paralyzing most of the industrial sectors. In response to this COVID-19 outbreak, we summarize the impact of COVID-19 on all aspects of Pakistan's economy (30). Although in terms of mortality, COVID-19 does not have a similar pattern compared with the 2002–2003 severe acute respiratory syndrome (SARS), and its global spread is different from the Spanish Flu pandemic, which appeared in 1918–1919 (31).

The World Health Organization published survey results based on 130 countries in October 2020 to record the adverse influence of the virus (COVID-19) on various mental health issues. Almost 30% of countries encountered difficulties due to a lack of health workers to fight against the ongoing COVID-19 virus. Nearly 19% of member states of the WHO faced mental health issues. Of the member states, 28% had insufficient personal protective and preventive equipment. Conversely, 89% of the WHO member countries initiated protective measures and included psychological and mental support in the national preventive plans to combat the pandemic (32). The WHO report evidenced that monumental effects had monumental impact on global communities' mental health and universal well-being of societies. Visibly, due to insufficient capacity in responding to the COVID-19 outbreak, it is uncertain how this world will deal with the current looming disaster of mental health and global economic crisis. The COVID-19 disease quickly instigated substantial disruption to human societies, health care systems, and economies worldwide. The COVID-19 pandemic has caused global challenges and economic crises that are yet to unfold. This study examines the adverse impacts and strategic retorts on protective measures to combat the consequences of COVID-19 on social, environmental, economic, and health sectors worldwide. This article primarily aims to examine challenges, economic crises, and their effects on business activities, pressure on healthcare systems, and government support to revive society's normal state.

**Figure 1:**
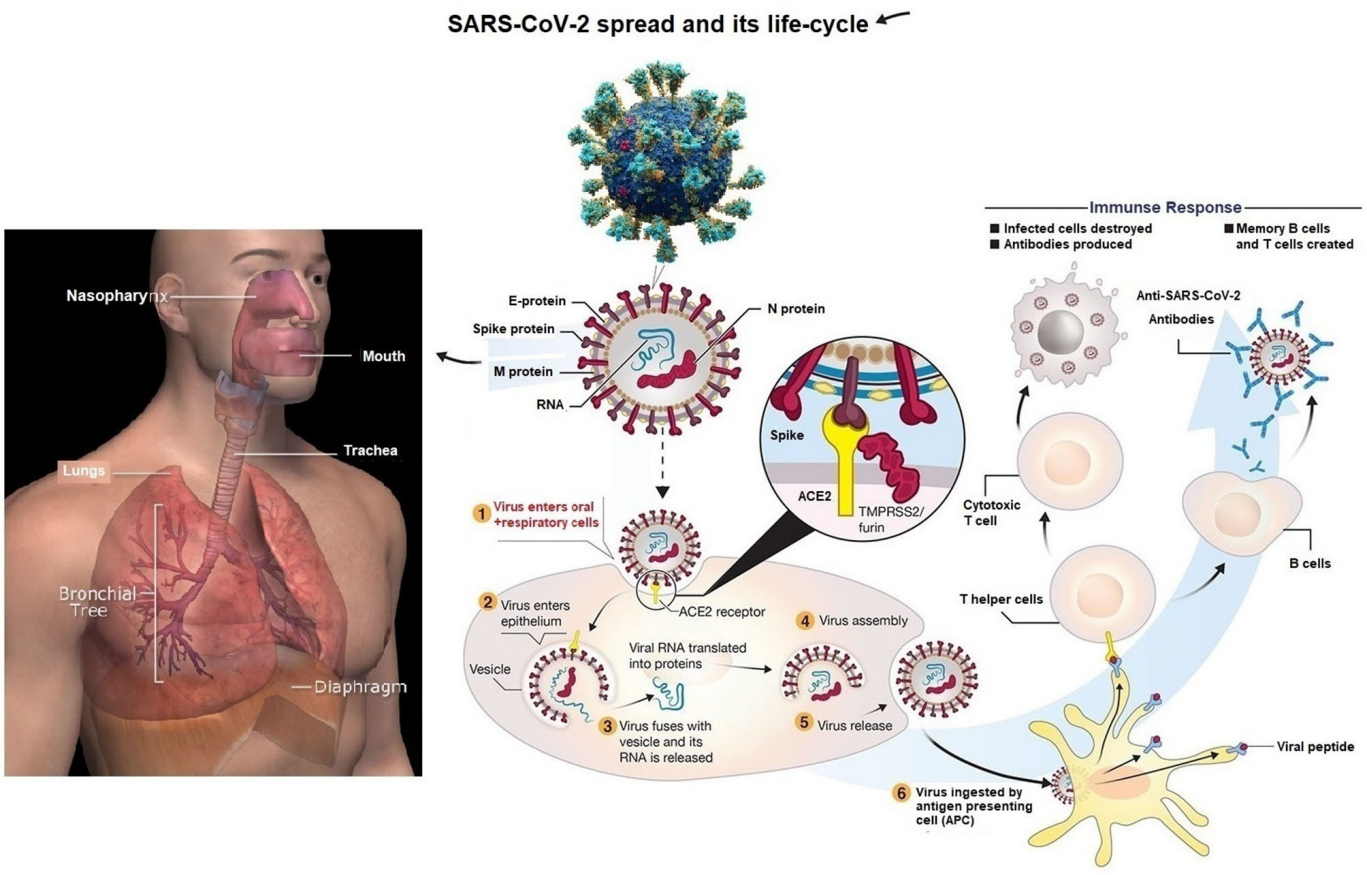
It shows SARS-CoV-2's spread and life-cycle, causing the contagious coronavirus disease. Source: the WHO data related to the COVID-19 pandemic.

Figure 1 specifies the life cycle and transmission of the virus, which causes the infectious disease COVID-19 (33). The virus transmits through respiratory droplets of coronavirus patients to oral and respiratory mucous membranes cells. Moreover, coronavirus disease possesses the single-stranded RNA genome enfolded in (N) nucleocapsid protein (the capsid together with the nucleic acids of a virus) and three main protein surfaces. These are enveloped (E), membrane (M), and Spike, which replicate and reach the lower airways and potentially cause severe pneumonia (33). Figure 1 shows the life cycle of the transmissible virus with its potential immune reactions.

The Health Ministry reported a 95.3% recovery rate based on 302,708 cases in Pakistan, with 317,595 total confirmed cases and 6,552 deaths with 2.12% fatality, which is much lower than the global average rate (3.6%). The COVID-19 virus has caused over 36.84 million positive patients and 1,067,560 deaths. There are over 27.70 million patients who had fully recovered from this infectious virus by October 10, 2020 worldwide (34). Table 1 shows the top 10 countries based on recovery, as of November 05, 2020.

**Table 1:** Global total cases, recoveries.

**Sr**.	**Country**	**Total cases**	**Total deaths**	**Total recoveries**	**Active cases**	**Critical cases**	**Total tests**	**Total population**
	**Worldwide**	**48,994,297**	**1,238,736**	**34,955,294**	**12,800,267**	**89,961**	**Country**	
1	USA	9,910,354	240,900	6,334,775	3,334,679	18,074	153,384,972	331,679,824
2	India	8,411,034	125,029	7,764,763	521,242	8,944	114,208,384	1,384,715,664
3	Brazil	5,614,258	161,779	5,064,344	388,135	8,318	21,900,000	213,085,794
4	Russia	1,712,858	29,509	1,279,169	404,180	2,300	63,000,000	145,956,429
5	France	1,601,367	39,037	124,278	1,438,052	4,230	17,055,801	65,324,114
6	Spain	1,365,895	38,486	150,970	N/A	2,802	18,072,174	46,761,136
7	Argentina	1,217,028	32,766	1,030,137	154,125	4,713	3,142,837	45,339,899
8	UK	1,123,197	48,120	N/A	N/A	1,191	35,241,533	68,010,512
9	Colombia	1,117,977	32,209	1,011,166	74,602	2,376	5,258,238	51,071,028
10	Mexico	943,630	93,228	697,402	153,000	2,838	2,445,709	129,402,754

*Top 10 countries summary, as of November 5, 2020. Source: COVID-19 CSC data, JHU*.

In the current situation, the incubation period of the COVID-19 is prevailing. However, the spread has reached almost every country worldwide. Health professionals have recommended self-isolation and social distancing by restricting social gatherings in cities and remote areas to stop the rapid spread. Smart lockdown measures are helpful to minimize the risk of large-scale infection spread of COVID-19. Avoiding social distancing would overwhelm the healthcare systems, causing massive scale causalities of human lives. However, blockades often choke economic development in numerous ways (35). The lockdown strategy helps control the rapid spread of the COVID-19 infection; however, it leads to adverse financial, health, and social factors. The COVID-19 pandemic has created the worst blockade, which has resulted in job losses of 5 million in Pakistan. The crisis of coronavirus (COVID-19) has taken a more massive toll on job losses than previously feared. The jobs and income losses have led to more hunger and had adverse effects on individuals' quality of life. The pandemic has developed to be a severe threat. Table 2 provides detail.

**Table 2:** COVID-19: case mortality analysis worldwide (February 20, 2021).

	**Country**	**Confirmed**	**Deaths**	**Case- fatality**	**Deaths/100 k pop**
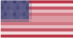	United States	27,896,042	493,082	1.8%	150.71
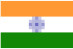	India	10,963,394	156,111	1.4%	11.54
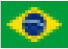	Brazil	10,030,626	243,457	2.4%	116.23
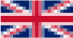	United Kingdom	4,095,187	119,614	2.9%	179.90
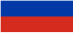	Russia	4,079,407	80,587	2.0%	55.78
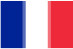	France	3,596,156	83,542	2.3%	124.71
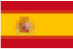	Spain	3,121,687	66,704	2.1%	142.76
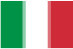	Italy	2,765,412	94,887	3.4%	157.02
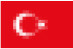	Turkey	2,616,600	27,821	1.1%	33.80
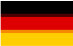	Germany	2,372,209	67,245	2.8%	81.09
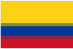	Colombia	2,212,525	58,334	2.6%	117.49
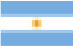	Argentina	2,046,795	50,857	2.5%	114.30
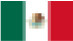	Mexico	2,022,662	178,108	8.8%	141.14
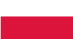	Poland	1,614,446	41,582	2.6%	109.49
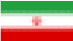	Iran	1,550,142	59,264	3.8%	72.45
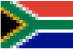	South Africa	1,498,766	48,708	3.2%	84.30
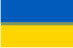	Ukraine	1,333,332	26,191	2.0%	58.69
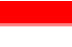	Indonesia	1,252,685	33,969	2.7%	12.69
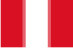	Peru	1,252,137	44,308	3.5%	138.51
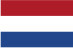	Netherlands	1,057,116	15,211	1.4%	88.28
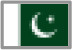	Pakistan	568,506	12,527	2.2%	5.90

*Source: COVID-19 CSC data, JHU*.

**Figure 2:**
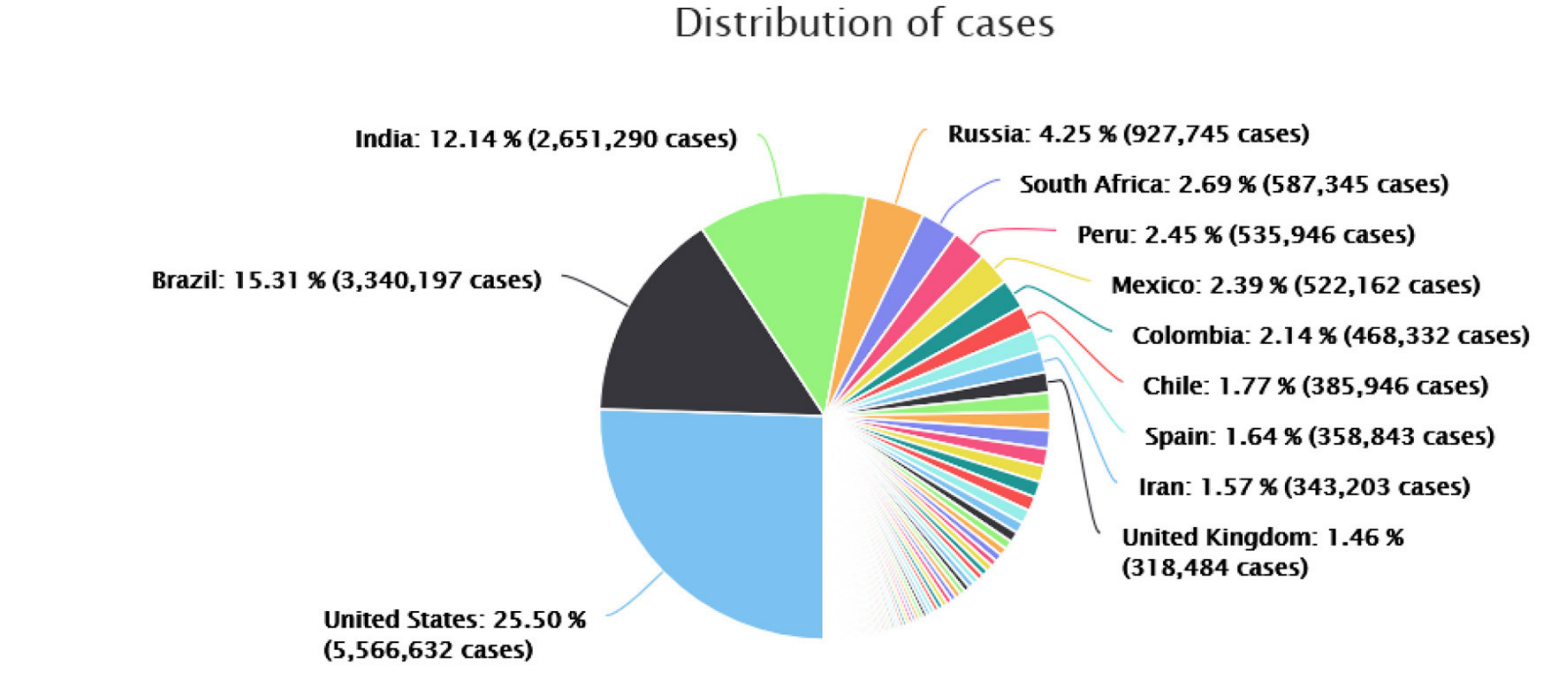
COVID-19: analysis by countries with highest global cases (August 16, 2020). Source: data provided by World Health Organization related to coronavirus (COVID-19).

Table 2 displays COVID-19 positive cases in countries with higher numbers of patients worldwide. Figure 2 specifies that the US is the most affected country with 25.5% of global cases in terms of confirmed COVID-19 patients (5,566,632), followed by Brazil 15.31% (3,340,197), India 12.14% (2,651,290), and Russia 4.25% (927,745). South Africa has 2.69% (587,345), Peru 2.45% (535,946), and Mexico 2.39% (522,162), Colombia showed 2.14% (468,332), Chile 1.77% (385,946), Spain 1.64% (358,843), Iran 1.57% (343,203), and the United Kingdom 1.46% (318,484). Table 3 shows countries reporting the highest death toll worldwide, as of February 20, 2021.

**Table 3:** COVID-19: case mortality analysis - deaths worldwide (February 19, 2021).

**Country**	**Confirmed**	**Deaths**	**Case-Fatality**	**Deaths/100 k pop**.
United States	27,896,042	493,082	1.8%	150.71
Brazil	10,030,626	243,457	2.4%	116.23
Mexico	2,022,662	178,108	8.8%	141.14
India	10,963,394	156,111	1.4%	11.54
United Kingdom	4,095,187	119,614	2.9%	179.90
Italy	2,765,412	94,887	3.4%	157.02
France	3,596,156	83,542	2.3%	124.71
Russia	4,079,407	80,587	2.0%	55.78
Germany	2,372,209	67,245	2.8%	81.09
Spain	3,121,687	66,704	2.1%	142.76
Iran	1,550,142	59,264	3.8%	72.45
Colombia	2,212,525	58,334	2.6%	117.49
Argentina	2,046,795	50,857	2.5%	114.30
South Africa	1,498,766	48,708	3.2%	84.30
Peru	1,252,137	44,308	3.5%	138.51
Poland	1,614,446	41,582	2.6%	109.49
Indonesia	1,252,685	33,969	2.7%	12.69
Turkey	2,616,600	27,821	1.1%	33.80
Ukraine	1,333,332	26,191	2.0%	58.69
Belgium	746,302	21,821	2.9%	191.04
Pakistan	568,506	12,527	2.2%	5.90

*Source: COVID-19 CSC data, JHU*.

Table 3 presents the countries reporting the highest deaths caused by the COVID-19 pandemic worldwide. The US has the highest number of COVID-19 confirmed cases (27,896,042) and deaths (493,082) with a case-fatality ratio of 1.8%. Brazil is the second highest state with established patients of COVID-19 (10,030,626), deaths (243,457), and case-fatality rate (2.4%). Mexico is the third most affected country from the COVID-19 pandemic with high confirmed infected cases (2,022,662), deaths (178,108), and case-fatality ratio (8.8%).

**Figure 3:**
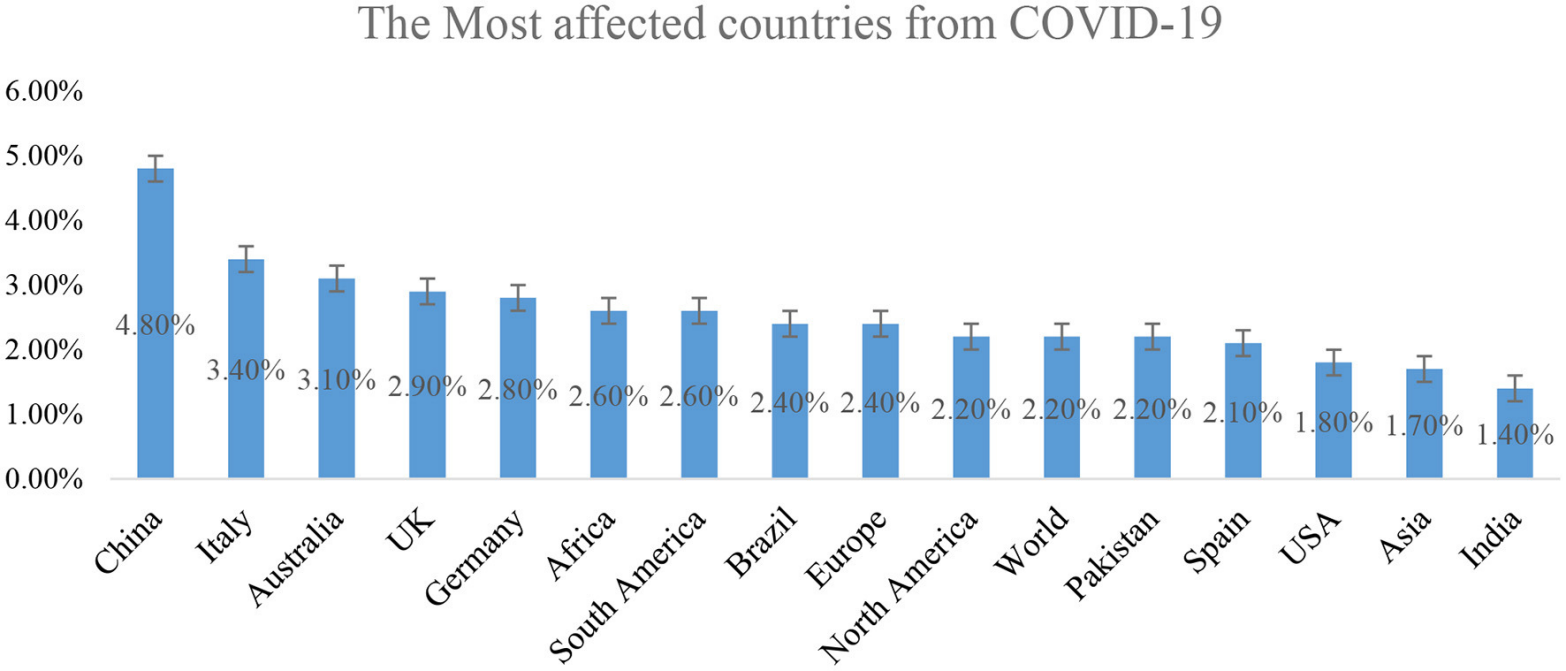
COVID-19 Case-fatality-rate (CFR) worldwide - February 15, 2021.

Figure 3 exhibits global CFR (Case-fatality-ratio) of the COVID-19 pandemic. China reported a 4.8% case fatality ratio, Italy 3.4%, Australia 3.1%, the United Kingdom 2.9%, Germany 2.8%, and Africa 2.6%. Similarly, South America reported 2.6%, Brazil 2.4%, Europe 2.4%, North America 2.2%, the world 2.2%, Pakistan 2.2%, the US reported 1.8%, and Asia recorded 1.7% CFR ratio for the COVID-19 virus.

**Figure 4:**
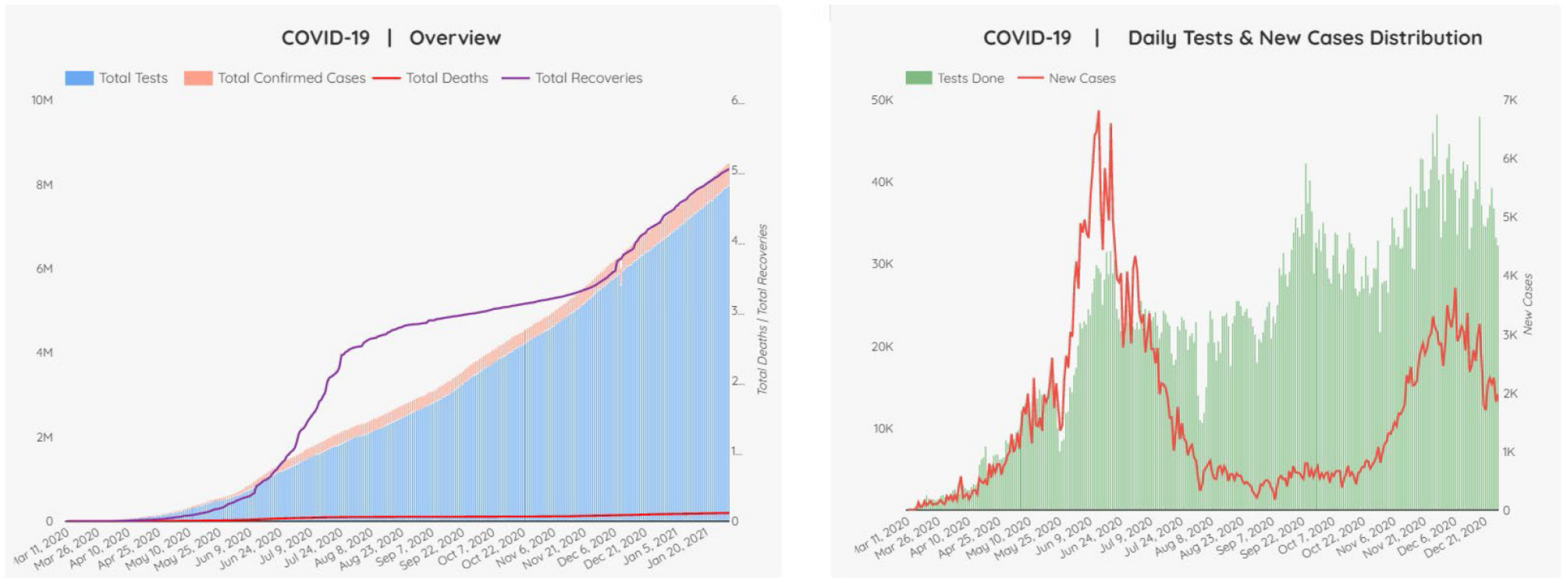
COVID-19 across Pakistan - February 12, 2021. Source: Ministry of Health, Pakistan.

Figure 4 indicates the overall scenario of the COVID-19 pandemic in Pakistan. Figure 4 shows the COVID-19 overview with daily new tests and new cases' distribution in Pakistan. The lockdown strategy helps control the rapid spread of the COVID-19 infection; however, it leads to adverse financial, health, and social factors. COVID-19 has created the worst blockade, which has resulted in job losses of 5 million in Pakistan. The crisis of coronavirus (COVID-19) has taken a more massive toll on job losses than previously feared. The jobs and income losses have led to more hunger and left adverse effects on individuals' quality of life. The pandemic has developed to be a severe threat to the lower-income groups of society (36). It is vital to initiate measures to control the spread of the epidemic without destroying economic growth. The financial experts advised to eliminate adverse elements of the COVID-19 on the economy and take measures to revive the industrial process through a smart lockdown strategy (37).

## The Nexus Between COVID-19 Arrival, Unemployment, and Decline in Economy

The economic experts estimated that Pakistan's economy might shrink by 15 billion US dollars in response to the adverse consequences of the COVID-19 pandemic. According to experts' prediction, the 4th quarter can show a 10% decline in Pakistan's GDP growth in the fiscal year of 2020. The government imposed full and smart lockdowns from March to June 2020, which resulted in no actual development of gross domestic product, and it reflected a negative (−2.0% GPD) growth in 2020, which would carry on to the first quarter of 2021. According to the Gallup Pakistan survey's findings, the unemployment rate might surge to a whopping 28%. There would be 6.65 million unemployed people during the fiscal year 2020–21, compared to 5.8 million in the preceeding year of 2020. Experts estimated that there would be a 30% to 40% layoff because of prolonged full or partial lockdowns, which has resulted in losses of 190 billion in the private sector (38). Before the arrival of the COVID-19 pandemic, the interest rate was over 13%. The State Bank of Pakistan dropped the interest rate to 7% on bank deposits in Mid-June 2020 to empower the business industry. However, a lower rate of return on investments in financial institutions has affected ordinary people as they were running their home expenses on the income of their deposits. The lower profit rate on deposits has caused mental stress for people who relied on their financial investments. Price-hike has increased, which resulted in lower spending power. Table 4 presents GDP, per capita income, and annual percentage change in growth.

**Table 4:** GDP, Per capita income, and annual %-change in growth.

	**GDP nominal**	**Per capita**	**Growth**
**Year**	**GDP (billions of US $)**	**Per capita (US $)**	**Annual % change**
2015	270.556	1,356.6678	4.731
2016	278.655	1,368.4543	5.527
2017	304.567	1,464.9933	5.554
2018	314.568	1,482.3057	5.836
2019	278.222	1,284.702	0.989
2020	262.000 e	1,200.00 e	−5.000*

* *By 2nd Quarter of 2020, e = expected estimation*.

## Economic Relief Package for Improving Quality of Life

The prime minister emphasized improving the quality of life of the public and approved an economic relief package of Rs. 2.1 trillion on March 14, 2020. The Government allocated one hundred fifty billion (Rs.150) for lower-income individuals, mainly laborer classes, and allotted PKR 280 billion (1.76 billion USD) for wheat procurement (27). The government ordered to defer interest payments of loans temporarily to support exporters and released Rs. 100-billion for subsidiaries to the agriculture sector along with small industries. The relief package offered a significant reduction in the prices of petroleum, electricity, and gas bills and postponed payments to pay later in installments, which helped to improve the quality of life of the public (39). Under this relief package, the government increased 50% of the Benazir Income Support Programme (BISP) to enhance the quality of life of the lower-income groups. Besides, 5.2 million beneficiaries through the BISP's ongoing National Socio-Economic Registry (NSER) and officials included more people in the package (40).

The Government also included health professionals in this relief support, as officials stated that doctors or paramedical staff die while treating the patients of COVID-19. The state would regard health professionals as martyrs, and their families would receive the same packages of the martyrs. The federal cabinet members reviewed and approved the economic package on March 13, 2020, to make a better quality of life for ordinary people (25). The Government established the Economic Coordination Committee to approve the relief package and granted supplementary Emergency Relief Fund of Rs. 100-billion to support 12 million low-income families in combating the COVID-19 pandemic. The Government provided cash assistance for 4 months to deserving families as a one-time dispensation after biometric verification and approval of the district administration under the Ehsaas Program. The Government had disbursed Rs. 22.466-billion among 1.77 million people as of April 15, 2020 (41).

China reported this contagious disease (COVID-19) for the first time in Wuhan, which infected people severely; however, the Government effectively controlled the illness within 5 months. Other successful countries, such as Singapore, South Korea, Pakistan, Australia, and New Zealand, also managed the rapid spread of this disease, and the trend of new cases has declined sharply. COVID-19 hit the United States, Spain, Italy, Spain, and France extremely hard at the start and put a heavy burden on their healthcare systems. It has developed to be a global health emergency more disastrous than the Second World War (37). As the developed countries around the world bear the brunt, the disease has also hit developing and emerging countries massively. Pakistan is on the brink of extinction, with tight ropes in the face of economic weakness. Next year is likely to be the most severe challenge Pakistan will encounter, which requires resilience, competence, and discipline. If the country fails, the other end of the equation is worse than our darkest nightmare (31). By 2019's second half, the world economy entered into a turbulent and challenging recession scenario. However, financial experts hoped economic conditions would improve in 2020 as large emerging economies came forward to lead the global economy back to potential economic growth by 2021. With the devastating effects of the COVID-19 eruption, all bets disappeared and revised all global growth forecasts as this pandemic has changed those growths downwards.

## The COVID-19 Pandemic's Adverse Impacts on Global Economies

The emergence of COVID-19 has negatively affected the global economy's economic growth beyond anything experienced within the past 100 years. The economic experts estimated that the COVID-19 pandemic could trim 3.0–6.0% off global economic growth by the end of 2020 (42). The experts expect a partial recovery of the global economy in 2021, assuming that there is no second wave of the spread of the pandemic. The spread of the infection has slowed down economies, and as a result, Pakistan is facing an economic, social, and health crisis and trying to revive its economic growth to improve individuals' quality of life. The adverse effects are severe, and GDP has shown negative growth for the first time in the last 60 years. The result is worsening in the current and financial balance, supply chains disruptions, and growing unemployment with job losses of 5 million. In January 2020, WHO professed the spread of the coronavirus as a world health emergency. Since then, the arrival of the pandemic (COVID-19) emergency has led to a global economic, social, and public health crisis, resulting in the loss of 90 trillion USD to global economies. Globally, governments have initiated steps to balance often-competing economic policy objectives to address the social and public health crisis with economic considerations to revive economic growth to stabilize their economies.

## The COVID-19 Pandemic Influence on Gross Domestic Product (GDP of Pakistan)

The fragile economy of Pakistan was already struggling to move toward a stable stage when the pandemic struck. The COVID-19 epidemic struck the economy massively. The financial experts estimated that economic fallout caused by COVID-19 would considerably derail the recovery process of the economy in Pakistan, which has already taken a devastating blow on economic growth (27). The pandemic has struck all the sectors, and the Pakistani economy has shrunken with raised unemployment due to the loss of 12.3 million jobs (11, 12). The growth of GDP was 5.8% in 2018, now GDP is 0.98%, and it is indicating a further decline for the coming years. The country's fiscal deficit is roughly 10.0%, and revenues of Pakistan have plummeted during the past 2 years. These indicators have specified that the appearance of the COVID-19 pandemic will seriously negatively affect the country (43).

## COVID-19 and Burden on Public Health System

The advent of the COVID-19 epidemic affected individuals' lifestyles around the world. Health education and counseling model is helpful to reduce mental stress and hypertensive disease in crisis situations (44). The outbreak has extensively changed healthcare demands and ways medical services are deliver to people, especially in Southeast Asia and Pakistan (45). The region has introduced digitalization in the health care systems. The outbreak of COVID-19 has shifted care from face-to-face physician's consultation to remote consultations through phone and online mediums. The coronavirus pandemic has caused immense challenges to the health care systems, people's lives, and the global economy (25). With the COVID-19 epidemic on the rise, society started to pay more attention to pharmaceutical industries and healthcare systems to provide better medical services, which had both negative and positive impacts across various sub-sectors. In the short-term, the pandemic caused a variety of consequences on the pharmaceutical industry, healthcare institutions, distribution and retail channels of pharmaceutical companies, and health insurance companies worldwide. Concerning the medium-term and long-term impacts, the COVID-19 pandemic effects on pharmaceutical companies and healthcare would be relatively positive (46). The healthcare system was already overstretched before the onset of the outbreak of the COVID-19 epidemic. The Pakistani health care system statistics specified that one doctor is available to treat 963 individuals, and there is one bed for 1,608 people, according to UNDP statistics. Pakistan received a bailout package of six billion US dollars from the IMF to tackle the monetary crisis. The Pakistani economy was progressing toward a stable phase to recover from the economic crisis when COVID-19 struck (47). The pandemic hit caused 12.50 million people to suffer from food security. One-third (35%) of the population was living below the poverty line before the epidemic, and now it is over 50% (48). At present, 66% of the population (150 million) are facing poverty, and they need immediate economic relief to improve their quality of life (Table 5).

**Table 5:** COVID-19 cases in selected Asian countries with patients victim by the virus.

**Country**	**Total**	**New**	**Total**	**New**	**Total**	**Active**	**Tot Cases/**	**Deaths/**	**Total**	**Population**
	**Cases**	**Cases**	**Deaths**	**Deaths**	**Recovered**	**Cases**	**1M pop**	**1M pop**	**Tests**	**Country wise**
Asia	59, 428, 348	206, 088	852, 867	3, 165	56, 039, 057	2, 536, 424				Country
India	31, 215, 142	42, 123	418, 511	489	30, 383, 001	413, 630	22, 389	300	447, 341, 133	1, 394, 235, 023
Turkey	5, 546, 166	8, 780	50, 650	46	5, 395, 300	100, 216	65, 027	594	65, 300, 191	85, 290, 746
Iran	3, 576, 148	27, 444	87, 624	250	3, 168, 834	319, 690	42, 013	1, 029	24, 892, 912	85, 119, 790
Indonesia	2, 950, 058	38, 325	76, 200	1, 280	2, 323, 666	550, 192	10, 667	276	23, 719, 489	276, 556, 785
Philippines	1, 517, 889	4, 502	26, 842	56	1, 444, 215	46, 832	13, 661	242	16, 086, 481	111, 109, 628
Iraq	1, 510, 517	8, 922	17, 951	59	1, 372, 158	120, 408	36, 693	436	12, 587, 768	41, 166, 304
Bangladesh	1, 128, 889	11, 579	18, 325	200	951, 340	159, 224	6, 784	110	7, 339, 909	166, 410, 269
Pakistan	993, 872	2, 145	22, 848	37	921, 095	49, 929	4, 410	101	15, 484, 282	225, 380, 778
Malaysia	939, 899	12, 366	7, 241	93	798, 955	133, 703	28, 655	221	16, 622, 925	32, 800, 653
Japan	843, 856	2, 171	15, 060	12	801, 337	27, 459	6, 694	119	17, 614, 879	126, 069, 343
Jordan	762, 420	392	9, 916	6	744, 365	8, 139	73, 954	962	8, 265, 981	10, 309, 372
Nepal	670, 953	2, 202	9, 607	25	634, 927	26, 419	22, 602	324	3, 486, 354	29, 685, 410
UAE	664, 027	1, 541	1, 904	4	641, 750	20, 373	66, 299	190	63, 206, 621	10, 015, 675
Saudi Arabia	512, 142	1, 273	8, 103	14	493, 240	10, 799	14, 475	229	23, 936, 986	35, 381, 779
Kazakhstan	499, 111	4, 889	5, 062	65	433, 994	60, 055	26, 252	266	11, 575, 012	19, 012, 164
Thailand	426, 475	11, 305	3, 502	80	296, 208	126, 765	6, 094	50	8, 129, 670	69, 983, 960
Kuwait	387, 912	1, 043	2, 247	9	369, 737	15, 928	89, 448	518	3, 264, 442	4, 336, 739
Sri Lanka	287, 973	1, 554	3, 870	43	262, 828	21, 275	13, 389	180	4, 195, 281	21, 507, 531
Bahrain	268, 027	78	1, 380	2	265, 832	815	152, 009	783	5, 296, 765	1, 763, 227
Qatar	224, 510	108	599	3	222, 362	1, 549	79, 959	213	2, 276, 171	2, 807, 805
S. Korea	180, 481	1, 278	2, 059	1	160, 347	18, 075	3, 517	40	11, 251, 987	51, 315, 548
Afghanistan	142, 800	386	6, 295	29	90, 153	46, 352	3, 584	158	683, 555	39, 845, 072
Uzbekistan	120, 631	658	805	4	115, 514	4, 312	3, 550	24	1, 377, 915	33, 977, 417
China	92, 342	65	4, 636	0	87, 098	608	64	3	160, 000, 000	1, 439, 323, 776
Maldives	76, 188	169	217	1	73, 340	2, 631	138, 395	394	1, 115, 829	550, 511

***Source:** COVID-19 CSC data, JHU. Yellow color shows new cases of Covid-19 and RED color indicates new deaths in various countries*.

*Table 5 indicates the situation of the selected Asian countries that have faced the hardest hit of the COVID-19 outbreak as of July 17, 2021. Table 4 shows the countries reporting the highest registered patients with deaths caused by the COVID-19 pandemic. India has reported the highest number of patients of the viral disease COVID-19 and reported confirmed cases (31,215,142) and deaths toll (418,511) with a CF (case-fatality) ratio = 1.3%. Turkey is the second most affected country from the pandemic and announces confirmed patients of COVID-19 (5,546,166) and total deaths (50,650) with a CF (case-fatality) ratio = 1.3%. Iran is the third most victim country from this viral disease COVID-19 and declared confirmed patients (3,576,148) and total deaths (87,624) with a CF ratio = 2.5% (case-fatality) as of July 19, 2021. See Figure 3*.

## Economic Indicators Under COVID-19 Pandemic

Risk analyst expert estimated that Pakistan's economy would shrink by 15 billion US dollars because of the COVID-19 pandemic. According to his prediction, the fourth quarter would indicate a 10% decline in Pakistan's GDP during the fiscal year of 2020. Complete or smart lockdowns would result in no actual growth of gross domestic product, or a negative −2.0% GPD growth in 2020, leading to the first quarter of 2021. Gallup Pakistan conducted research and reported that the unemployment rate would surge to a whopping 28%. During the fiscal year 2020–21, estimations indicated that unemployment would increase to 6.65 million, compared to 5.8 million in the preceding year of 2020. Experts also projected a 30–40% layoff in the formal sector because of prolonged complete or partial lockdowns, resulting in an impact of 190 billion in the private sector (38). Before the outbreak of COVID-19, the interest rate was over 13%, and the State Bank of Pakistan cut interest rates to ease borrowers by decreasing 225 basis points (7%) in Mid-June 2020, to empower the business industry. The experts indicated that Pakistan would need financial assistance from the IMF, the World Bank, or its strategic allies, such as China or other countries. Because of limited financial capacity to beat COVID-19, Pakistan would need more assistance to combat the adverse effects of the pandemic. According to the 2019 estimation, the total GDP of Pakistan was $284 billion (nominal), with 3.30% economic growth. However, it showed a negative growth rate of −2.60% after the pandemic. The COVID-19 epidemic caused a 10% decline to GDP with an estimated loss of Rs. 1.10 trillion in 2020. If exports fell by 20%, the Pakistan economy might face a 4.64% decline (49).

The Asian Development Bank stated that the global health emergency had declined growth to 2.20 in Asia, and it would rebound to 6.20 in the fiscal year 2021. In this challenging situation, innovation is crucial to gain inclusive, environmentally sustainable economic growth. Some developing economies in Asia are close to the global innovation frontier, while other countries lag behind. The Asian Development Bank approved a loan of 300 million US dollars to strengthen Pakistan's public health sector to combat the effects of the COVID-19 pandemic. It helped to meet the basic needs of the poor and vulnerable segments of society. ADB initiated the CARES program to help the Pakistan Government deliver social protection programs to vulnerable and lower-income groups of poor segments and expanded the health sector's capabilities. It provided a pro-poor fiscal inducement to boost economic growth and offered jobs to fight the adverse economic effects of COVID-19. AIIB facilitated parallel finance of 500 million US dollars to the CARES program, and it received another 500 million US dollars from the World Bank to support Pakistan. These measures helped Pakistan to improve the quality of life for the lower-income segments (38).

## The COVID-19 Appearance and Pakistan's Imports

Pakistan's total imports as of March 2020 were Rs. Five hundred twenty-five (Rs. 525) billion rupees, with a downtrend of −18.7% year-on-year, claimed by Pakistan Bureau of Statistics (PBS). According to the National Bank of Pakistan (SBP), the largest import partners were China, the United Arab Emirates, Singapore, the United States, and Saudi Arabia, accounting for 51% of total imports between July 2019 and February 2020. During that period, China alone contributed 21% to total imports (50, 51). Hence, imports decay indicates the effects of a series of factors, including supply chain disruptions, falling demand in Pakistan, and lower commodity and commodity prices (52). The fall in imports indicated a positive move toward Pakistan's current account deficit. PIDC2 described that Pakistan's 32% of imports determine final products that do not directly influence Pakistan's GDP. Further, raw materials commodities account for 68% of Pakistan's imports, such as intermediate and capital goods that refer to essential goods and raw materials to produce end commodities for domestic consumption and export. As a result, these declines will have a negative impact on investment spending and exports. Because of this, Pakistan may experience a knock-on effect of a decrease in imports, which will affect GDP. Hence, the decline in imports would lead to a negative impact on investment spending as well as exports. Pakistan could experience a chain reaction of falling import volume, which will negatively affect the country's gross domestic production (GDP). The import volume during 2020 was PKR six hundred fourteen thousand nine hundred thirty-four (614,934) million in July 2020, Rs. 611,449 million in June. It remained at Rs. 589,739 million in July 2019, which showed an increase of 0.57% increase in June 2020 and 4.27% in July 2019. The Balance of Trade always remains negative, and the average was Pak Rs. −46,486.18 Million from 1957 to till 2020, which means Pakistani spends more on imported items and earns less from exported commodities. It affects spending on public health to improve the quality of life (53, 54).

## The COVID-19 Outbreak and Exports Hurdles

Pakistan's total exports in March 2020 amounted to PKR 287.70 billion indicating a downward trend of 12.9% from the previous month, according to the Pakistan Bureau of Statistics (PBS). The most prominent export partners of Pakistan are the United States, the United Kingdom, China, Germany, and the Netherlands, which account for about 40% of the total exports, according to the central bank, State Bank of Pakistan (SBP). The outbreak of COVID-19 massively affected all the partner countries, which has adversely affected global trade. Worldwide, business and trade are showing a declining trend, and it will lead to a decrease in global trade activities as the trade demand has decreased since the coronavirus pandemic. Almost every country restricted social set ups and gatherings and imposed complete or a partial/smart lockdown, and closed public transportation in most situations. It has had an extreme effect on industry, and it disrupted the process of commodities' production (53, 54). According to the Pakistan commerce ministry's estimation, exports fall could reach 20% with the strike of the COVID-19 pandemic, and it could lead to a loss of 4 billion US dollars by June 2020 (52). In the post-COVID-19 world order scenario, Pakistan can take advantage of two emerging potential opportunities to reform its economy. The major competitors' economies of India and Bangladesh are squeezing drastically, and import commodities have become cheaper for the time being. Pakistan expects a roughly 20% fall in exports and remittances, and smart economic strategies can help to take commercial benefits by designing global supply chains. Pakistan can also exploit the tense trade situation between China and the United States and tailor strategies to attract foreign investors. The recession and economic crisis worldwide offers economic opportunities for Pakistan due to its important geopolitical location in the region. Pakistan can take up global orders to increase the export volume. It would help Pakistan to spend more on public health systems to improve the quality of life of the people.

## Remittances

Remittances from Pakistan were $1.824 billion in February 2020, which indicated a decreasing trend (−4.4%) from the previous month, according to the announcement of State Bank of Pakistan (SBP). The reason for the decline was the spread of the COVID-19 pandemic around the world. The remittances maintained a downward trend in May 2020. Overseas Pakistanis typically send remittances to Pakistan from oil export Gulf Cooperation Council countries. The largest share of remittances in February 2020 came from Saudi Arabia ($422 million), the United Arab Emirates ($387.1 million), and the United States ($333.5 million). According to the World Bank, remittances from 66 countries, particularly emerging and developing countries, accounted for more than 5% of GDP in 2019. In the case of Pakistan, remittances accounted for 7.7% of GDP in the same year. Pakistan received the highest ever remittances worth $2.768 billion with an increase of 12% for a single month in July 2020, as compared with June 2020. The statistics indicated a rise of 36.5% in remittances from July 2019. Analysts' reasons for rising remittances are primarily fewer pilgrims journeying to religious places. Overseas Pakistanis religious workers had higher savings, and travel restrictions and flight cancelation contributed to a rise in remittances. The Government facilitates overseas workers by improving channel efficiency and introduced incentives on foreign money transfers.

The World Bank emphasized that the outbreak of COVID-19 could severely affect remittances due to the closure of major countries. Financial experts have indicated a fall in remittances to Pakistan from major countries, like Saudi Arabia, UAE, and the United States. It is due to lockdown and blockade in these countries (49). From mid-June to August 2020, oversees Pakistanis contributed a lot to the Government's appeal by sending remittances with banking channels. It was the result of Pakistani schemes to attract overseas Pakistanis, which showed a tremendous positive impact on generating remittances inflows to Pakistan. The State Bank of Pakistan reported that overseas Pakistanis' remittances drastically increased to 50.7%, which reached sn all times best, worth 2.466 billion US dollars, as of June 30, closing the fiscal year 2020, with a closing total of 23.1 billion dollars compared with 1.636 billion US dollars in 2019. The remittances inflows to Pakistan remained steady and stable despite the hit of the COVID-19 epidemic that disrupted the global economy and caused unemployment and a substantial fall in remittances of the workers worldwide. The remittances inflows from July to December 2020 to May 2021 remained over 2 billion dollars with a consecutive 8-month increase in the remittances. Remittance inflow was reported at 2.27 billion in January 2021, which was 19% higher than January 2019 and 24% more than the last fiscal year. Pakistan can utilize these all-time high remittances to revive the economy and spending on public health to improve life quality (55).

## Linkage of Poverty and Unemployment on Life Quality

Pakistan's total workforce is 63.4 million, of which 26.41 million (41.6%) stands vulnerable, according to the Employment Trends reported by the Pakistan Bureau of Statistics in 2018. Vulnerable employment mentions a proportion of self-employed and workers employed domestically in total employment, including the poor class workers relying on daily wages. These workers are likely to be the most affected. They may lose their jobs because of the COVID-19 pandemic in Pakistan (56). The experts have warned of a significant increase in the number of poor and unemployed workers in the coming months in response to complete or partial lockdowns imposed by the Government to control the spread of the pandemic. It has slowed down economic activities, which leads to a high proportion of vulnerable employment in the country. PIDE released a report stating that the Government's modest restrictions could result in unemployment of 12.3 million workers, which represents 46.3% of the entire number of vulnerable employment and 19.4% of the whole employment number in Pakistan (57). According to the experts' estimation, the number of layoffs/job cuts in the retail and wholesale sectors would reach 4.55 million. As a result, Pakistan's poverty rate could rise from 23.40 to 44.20%, which will negatively impact individuals' quality of life (58).

## COVID-19 and Trade Disruptions

The emergence of a global health emergency caused by the hit of the COVID-19 epidemic challenged global trade. Globally, enterprises encountered numerous problems with certain levels of economic losses, such as fall in demand, raw materials shortage, and disruptions in trade, transportation, supply chains, and export commodities orders cancelations. The outbreak of COVID-19 massively affected micro as well as small and medium enterprises worldwide. These firms are the backbone and engine of global economies, which generate employment opportunities on a large scale. The Pakistani SMEs' contribution is substantial. They add 40% to national GDP, and 90% of the registered 3.2 million enterprises are SMEs and contribute over 40% of total exports (11, 12, 59). At the domestic level, social distancing measures, mainly the blockade, have caused inconvenience and shortages of supply. Besides, import restrictions and delays/cancellations of export orders have led to a decline in global trade, which has significantly slowed the pace of economic and trade activities. Even if defensive measures have contained the spread of coronavirus, the internal economic functions of the economy are facing disruptions; however, the COVID-19 pandemic has struck countries that are part of the global value chain (VGC) (35, 60). Pakistan imposed social gathering restrictions and ordered to close educational intuitions to minimize the rapid spread of the coronavirus disease through complete and smart lockdowns at various stages, which caused disruptions in economic and trade activities. The five main trading partners of Pakistan who account for 50% of the trade share are China, the United States, the United Kingdom, Japan, and Germany. Four of these partners are also the countries hardest hit by COVID-19. The pandemic of coronavirus has severely affected the international trade flows of these countries, and export volume from China and Japan fell over 15%. The other three countries' exports slowed down by 5% (61–63).

## The COVID-19 Pandemic Influences on Economy's Critical Sectors

The ongoing spread of the coronavirus pandemic affects the global economy. As a result, this epidemic (COVID-19) also hit all sectors of Pakistan economies, which disrupted trade and economic activities in the country. The crisis has affected the real estate market and property valuation and changed customer satisfaction in many industries (64–66). The pandemic is still spreading worldwide, and Pakistan's business community has begun to look for alternative options to purchase raw materials to produce goods. Spectrum Securities Limited released a report and warned that if the pandemic continues to spread for the next few months, it will adversely affect various sectors of the economy (67–70). The report stated that there is a prolonged interruption in the supply chains to receive raw materials. China has restricted transportation and other business activities to mitigate the virus infection. Accordingly, the industrial sector is facing a significant shortage of raw material supplies as they import these items from China. It has slowed down manufacturing activities, and further delays might have disrupted producing goods. This panic situation has caused more inflation and left adverse effects on critical accounts of the economic sectors. However, Pakistan reported an 11.4% upsurge in large-scale manufacturing by December 2020 over the previous year (36).

## Impact of COVID-19 Outbreak on the Steel Industry

The competition in the steel industry is very high, and producers tried to meet the global demand of 1,869.9 million tons of crude steel with a rising demand of 5.7%, with 1,341.6 Million tons production in and 3.4% more produced worldwide in 2019. China is the leading global exporter of steel with the highest output in the world. Pakistan imported steel products amounting to 1,390,561 million US dollars, which was 3% of the total steel exports in China in 2019. At the advent of the coronavirus (COVID-19), China's steel industry faced great difficulties due to lockdown and closure of the industry (71). In China and the rest of the world, the long-term effects of the coronavirus pandemic (COVID-19) are still pending. The experts have predicted that this outbreak has adversely struck the global steel industry, at least in the short to medium term. China is the largest producer of steel and its alloys, and it has restricted supplies and transportation. At the same time, India is focusing on increasing its share of steel products and raw materials goods in the global steel market (63, 72).

On the other hand, Pakistan's steel industry significantly relies on raw materials imported from China. Japan and the local industry are under pressure to produce goods because of this blockade from China. The effects of the infection of COVID-19 are still ongoing, and the business community is looking for alternatives at a reasonable cost to produce products to supply orders to carry on manufacturing and construction activities in the country. India is the second-largest steel producer after China, with an annual output of more than 106 million tons. Pakistan has the opportunity to buy raw materials for steel production from India. Over the past few years, excessive tensions between India and Pakistan have limited imports of various industrial raw materials, such as certain raw materials to produce pharmaceutical products. Pharmaceutical firms in Pakistan need to import raw materials to produce medicines to combat COVID-19 and provide relief to the public to improve the quality of life (73).

## Linkage Between COVID-19 and Decline in Tourism and Travel Industry

The advent of the infectious disease COVID-19 has hugely affected the tourism industry by bringing the world to a standstill position. Against a background of heightened uncertainty, reliable and up-to-date information is more imperative than ever before both for the tourism industry and tourists (74). The outbreak of COVID-19 has had a significant effect on tourism due to travel restrictions with a slump in travel demand among tourists worldwide. Several countries and regions have posed travel restrictions and entry bans to contain the spread of COVID-19 infection. The World Tourism Organization of UNO projected that international tourist arrivals would fall by 20–30% in 2020, which will lead to a potential economic loss of 30–50 billion US dollars. The global travelers' planned travel went down by 80–90% due to unilateral and conflicting travel restrictions that have taken place regionally due to the COVID-19 pandemic. The pandemic adversely affected tourist attractions, such as sports venues, amusement parks, museums, and entertainment worldwide. Many countries have restricted entries to main tourism destinations, airlines have restricted flight operations, organizers canceled business meetings and conferences, and hotels canceled room bookings. These measures against the spread of the pandemic have affected the tourism and hotel industry worldwide. Pakistan also posed restrictions on tourism and the hotel industry, and bookings have dropped drastically, which caused a significant loss to the hotel industry (75).

Many workers have lost their employment since March 2020. Numerous hotels received orders to cancel reservations as foreign travelers canceled travel plans, domestic tourists halted travel, and companies' business officials postponed travel activities. Pakistan Hotel Association stated that 200 registered business members with the hotel industry dropped their booking drastically with the advent of the pandemic in Pakistan. The hotel industry recorded a PKR 100 million loss in February 2020 due to a significant fall of guests. The emergence of the epidemic (COVID-19) has also affected the economy of Pakistan severely. There are 317,595 confirmed cases of coronavirus in Pakistan, as of October 9, 2020, and the pandemic has massively affected all sectors of the national economy. By January, the booking rate reached 95%, and it fell to 40% by the first week of March 2020 for the hotel industry (76, 77). With the reduction of outbound tourism, the amount of inbound tourism in Pakistan has also remained significantly reduced. Besides, tourism trends in Pakistan have fallen by 60–70% due to concerns about the coronavirus. The number of travelers leaving Pakistan for overseas travel is minimal, mainly due to the European embargo and the United States travel instructions issued to travelers from different countries. However, summer vacations were once the peak season for tourism in Pakistan. However, most businesses will now cease to close after an early leave announcement in Sindh this year (78).

## The Massive Impact of Coronavirus on Property Markets

The emergence of the COVID-19 pandemic has massively hit the real estate and energy sectors in unprecedented ways around the world (79–85). The unemployment rate in the United States remains sky high with the advent of the pandemic's lockdown and closure of business activities. The unemployment rate was 13.3% in May although down from 14.70% recorded in April 2020. If the pandemic prolongs, it will cause significant problems and possibly a knock-on effect. The capital value of the real estate's retail properties falls by 20–30%, according to Professor Nori Gerardo Lietz, a real estate investment teacher at Harvard Business School. The unlevered value of enterprises of the real estate assets had decreased by more than 25% in most sectors, particularly in the hotel and leisure sectors (86). The COVID-19 pandemic has hit the real estate sector in Italy, and estimation shown the fall in turnover between €9 and 22 billion compared with the first quarter of 2019. This result refers to the already rising demand for high-quality properties, which provide a safe, convenient, efficient, and healthy working and living environment. Concerning the real estate industry in Pakistan, it suffered a lot after a change in the government regime in 2018. The Government launched new policies and taxes, which greatly affected this industry and dampened consumers' confidence. The past 2 years immensely changed a large number of property dealers, builders, and investors because of unfavorable economic conditions. The real estate survives mainly on the significant investments by overseas Pakistanis; however, this test seems longer as the COVID-19 pandemic has hugely affected everyone and the real estate market in all major cities, including Lahore, Islamabad, Karachi, and Multan received a massive hit (87, 88). Bahria Town Lahore and Karachi closed all public spaces and closed places in these societies, including the Eiffel Tower, Zoo, Gymnasiums, Parks, and Sports fields. It means that all real estate transactions will stop for an indefinite period until the situation improves. The situation is getting better, and the real estate industry is reviving to reasonable conditions gradually as the Government has recently lifted travel restrictions (65). However, fear still remains. As a precautionary measure, some major builders of societies, including Bahria Town Lahore, Islamabad and Karachi, and DHA projects, have closed offices and facilities to mitigate the spread of the pandemic (87).

## The COVID-19 Adversity and Pharmaceutical Sector

Pakistan's pharmaceutical industry made good growth during recent decades (89–94). There are more than 800 large volume companies of pharmaceutical units, including 25 multinational operated pharma firms in the country (95). The pharma companies import raw materials from abroad to make medicines (91). The primary raw materials come from China, India, and Europe. The pandemic resulted in numerous health challenges (25, 29, 96–98). The Pakistan pharmaceutical industry is self-sufficient and meets more than 90% of the demand of the country. China is a significant producer of low-cost generic medicines and raw materials to make medicines (99). Most of the pharmaceutical companies entirely or partially rely on the pharmaceutical industry of China. Pakistan relies on China to supply active drug ingredients and other chemicals such as paracetamol, penicillin, analgesic ibuprofen, and popular diabetes drugs. However, pharma companies import some semi-APIs from India, Europe, and other countries. Imports from China account for 25% of Pakistan's total chemical and pharmaceutical raw materials to produce final drugs. While this critical figure could affect the country's drug production, it depends on the level of stock maintained by local pharmaceutical companies. Pakistan imports some portion of the medicines from overseas pharmaceutical companies. The advent of COVID-19 has massively affected the production of drugs worldwide. The outbreak of COVID-19 struck Pakistan and disrupted the supply chain of drugs. COVID-19 caused a shortage of raw materials and resulted in massive problems due to restrictions on transportation from China and other global suppliers (100). The crisis caused challenges for the people and affects life quality massively (43). Prices of medicines have risen, and it greatly affected the quality of life of the people (43, 101, 102). It was out of reach for people because of the economic crisis in Pakistan. Social support programs can secure workers health safety, which can increase employees' mental well-being (103–106).

## Discussion and Conclusion

The Coronavirus pandemic (COVID-19) emerged as the worst health calamity of the world within the past century. Globally, human societies faced the most thought-provoking health disaster since the catastrophe of World War II. Wuhan city reported this new type of infectious respiratory disease at the end of December 2019. The pandemic spread rapidly worldwide, posing substantial economic, environmental, social, and health challenges and massively disrupting social, economic, and religious communication and interaction worldwide (107). Globally, many countries are working to reduce the rapid spread of this ongoing COVID-19 pandemic through experimental facilities, identifying suspected and infected patients, and restricting social gatherings by implementing lockdown strategies (108). This study focused on identifying the massive effects of the COVID-19 epidemic on various segments of human society. The advent of the COVID-19 pandemic has shaken political, environmental, economic, health, and social factors foundations around the world. The pandemic caused mental stress and individuals used social media platform to seek health-related information. Some people were addicted to Facebook (44). At present, the appearance of the COVID-19 outbreak has most severely affected developing countries, including Pakistan. The pandemic has posed challenges for prioritizing lifestyle based on health promotion to prioritize health needs for students (109, 110). The healthcare systems, economic growth, resources, and governance problems have obstructed remedial action to revive the economy. This study aims to provide some limited prediction work on the economic impact of Covid-19 in the entire core economic field to illustrate the financial danger posed by the coronavirus pandemic to Pakistan. The ongoing spread of the coronavirus epidemic impacted economies. As a result, the (COVID-19) pandemic also hit the Pakistan economy's critical sectors and disrupted social interaction, trade, and economic activities. The government of Pakistan approved economic relief packages to uplift the lower-income segments of Pakistani society and provided financial support to improve their quality of life.

The pandemic spread has massively affected significant factors of the economy, such as imports, exports, remittance, public health, tourism, steel, agriculture, real estate, and pharmaceutical sectors. The adverse effects of the pandemic are still spreading, and business communities are searching for alternative ways to import raw materials for goods productions. Pakistani business firms mostly import raw materials from China, India, and other countries; however, China and other suppliers have posed restrictions on transportation and business activities to suppress the infection of COVID-19. The industrial sector has encountered a great challenge of raw material supply from China, which delayed the manufacturing process of commodities. The ongoing spread of COVID-19 caused a price hike and higher inflation and affected quality of life. The prices of medicines have risen, and it greatly affected the quality of life of the people. Medicine was out of reach for people because of the economic crisis in Pakistan. The arrival of the COVID-19 pandemic struck the tourism industry adversely and brought the world to a standstill position. Pakistan has reported an almost 11.4% increase in large-scale manufacturing by December 2020 over the previous year. Against a backdrop of heightened uncertainty and a dreaded situation of the coronavirus pandemic, reliable and up-to-date news is imperative for the tourism industry and tourists. Travel restrictions caused by the epidemic have adversely affected tourism and hotel industries with a slump in travel demand around the world. Numerous countries imposed travel restrictions as well as entry bans to control and mitigate infection of the coronavirus. With the blockade of economic activities, the pandemic spread has dramatically affected the quality of life of ordinary people worldwide. Evidently, due to insufficient resources to manage the adverse effects of the COVID-19 pandemic, it is uncertain how the health professionals and world leaders will deal with the present looming global mental health challenges and the COVID-19's spillover impacts on the economic crisis worldwide.

This study results provide a detailed analysis of the critical factors of the economy and health issues with global perspective implications. This article primarily focused on exploring economic consequences on the global economies and discussed Pakistan as a case study. The study contributes to the literature on the COVID-19 crisis. It examined the implications of the pandemic on financial and global health issues by examining America, Asia, Europe, Africa, Australia, and the Middle East. This study reports some limitations as it discussed health challenges and some critical economic factors that caused significant disruptions' with the downward trend of the economic growth. Forthcoming studies can explore other elements due to COVID-19 that posed damage to the economy and mental well-being. Future researches can investigate global health emergencies and disruptions in the mechanism of supply and demand. The effect that the pandemic has had on travel, service, tourism, food, and energy consumption demands can contribute interesting results. The pandemic's upcoming studies can explore the COVID-19 outbreak's socio-economic effects, available treatments, vaccination facilities, and reducing mental health stress to fight against this ongoing infectious disease. The study proposes non-pharmaceutical interventions to formulate smart lockdown strategies to restart economic activities. Work from home, online business, home delivery services, digital currency use, and Government support for health and business support will help progress toward the next normal in society.

## Data Availability Statement

The original contributions presented in the study are included in the article/supplementary material, further inquiries can be directed to the corresponding author/s.

## Author Contributions

JA conceptualized the idea, contributed to study design, completed the entire article, including introduction, literature, discussion, conclusion, and edited the original manuscript before submission. CW and KD contributed to edited the revised manuscript and contributed to the literature, discussion, and conclusion. DW the study design, analysis, reviewed and approved the final edited version, and supervised this research paper. RM approved the final edited version and contributed to the literature, discussion, and conclusion. All authors contributed to the article and approved the submitted version.

## Conflict of Interest

The authors declare that the research was conducted in the absence of any commercial or financial relationships that could be construed as a potential conflict of interest.

## Publisher's Note

All claims expressed in this article are solely those of the authors and do not necessarily represent those of their affiliated organizations, or those of the publisher, the editors and the reviewers. Any product that may be evaluated in this article, or claim that may be made by its manufacturer, is not guaranteed or endorsed by the publisher.
